# Sentinel lymph node biopsy in oral cavity cancer using indocyanine green: A systematic review and meta-analysis

**DOI:** 10.6061/clinics/2021/e2573

**Published:** 2021-07-08

**Authors:** Yongfeng Chen, Qi Xiao, Weina Zou, Chengwan Xia, Hongling Yin, Yumei Pu, Yuxin Wang, Kai Zhang

**Affiliations:** I Department of Stomatology, The First Affiliated Hospital of Bengbu Medical College, Bengbu, China.; II Department of Oral and Maxillofacial Surgery, Nanjing Stomatological Hospital, Medical School of Nanjing University, Nanjing, China.

**Keywords:** Oral Cavity Cancer, Indocyanine Green, Sentinel Lymph Node Biopsy, Sensitivity, Specificity

## Abstract

This meta-analysis was conducted to evaluate the value of indocyanine green (ICG) in guiding sentinel lymph node biopsy (SLNB) for patients with oral cavity cancer.

An electronic database search (PubMed, MEDLINE, Cochrane Library, Embase, and Web of Science) was performed from their inception to June 2020 to retrieve clinical studies of ICG applied to SLNB for oral cavity cancer. Data were extracted from 14 relevant articles (226 patients), and 9 studies (134 patients) were finally included in the meta-analysis according to the inclusion and exclusion criteria.

The pooled sentinel lymph node (SLN) sensitivity, specificity, positive likelihood ratio, negative likelihood ratio, and diagnostic odds ratio were 88.0% (95% confidence interval [CI], 74.0-96.0), 64.0% (95% CI, 61.0-66.0), 2.45 (95% CI, 1.31-4.60), 0.40 (95% CI, 0.17-0.90), and 7.30 (95% CI, 1.74-30.68), respectively. The area under the summary receiver operating characteristic curve was 0.8805.

In conclusion, ICG applied to SLNB can effectively predict the status of regional lymph nodes in oral cavity cancer.

## INTRODUCTION

Oral cavity cancer endangers human life and is the most common cancer worldwide. According to the National Comprehensive Cancer Network (NCCN) guidelines on oral cavity cancer treatment, cervical LN (lymph node) status is a critical determinant of therapeutic intervention. Selective neck dissection is performed for patients with undetectable LN or regional metastases, with a risk greater than 20% (1). However, cervical LN dissection affects the quality of life of the patients (2). Typical complications of this procedure include postoperative hematoma, wound dehiscence, chyle leak, skin flap necrosis, parotid gland fistula, and postoperative shoulder dysfunction (3). Therefore, accurate assessment of LN status in oral cavity cancer will help clinicians preserve patient function and reduce surgical complications as much as possible while thoroughly treating patients for their diseases.

Sentinel LN biopsy (SLNB) is an auxiliary diagnostic method for detecting and assessing neck metastasis and unpredictable lymphatic drainage in cN_0_ patients and has also been successfully applied in the detection of occult LN metastasis in head and neck cancer (HNC). Thompson et al. (4) concluded that the sensitivity of SLNB for the determination of regional LN metastasis was 94%. In addition, it was found that the negative predictive value (NPV) was 96% in 26 studies (766 patients with HNC). Civantos et al. (5) performed step sectioning and immunohistochemical tests on T1 or T2 patients (clinically N_0_) in oral squamous cell carcinomas, and the results showed that SLNB had a high accuracy with an NPV of 96%.

Indocyanine green (ICG) near-infrared (NIR) fluorescence imaging technology is also an SLNB technique that can detect sentinel lymph nodes (SLNs) favorably. This method has been clinically applied in the intraoperative imaging of lung cancer, breast cancer, and gastric cancer. However, the accuracy of ICG assessment and detection of SLN in oral cavity cancer is controversial because of the large number of LNs and the complicated drainage pathway, even with the possibility of skip metastases (6,7). Therefore, this systematic review and meta-analysis was performed to evaluate the diagnostic value of ICG for SLNs in oral cavity cancer.

## MATERIALS AND METHODS

### Search strategy

The protocol for the review was registered *a priori* in the International Prospective Register of Systematic Reviews under the registration number CRD42020134291.

An electronic database search (PubMed, MEDLINE, Cochrane Library, Embase, and Web of Science) was performed from the period of their inception to June 2020, which helped us retrieve clinical studies regarding the application of ICG in SLNB for HNC. The search strategy involved the use of the keywords “indocyanine green OR ICG” and “oral cancer OR gingiva cancer OR tongue cancer OR month floor cancer OR lip cancer OR buccal mucosa cancer OR palate cancer” and “lymph node OR LN OR lymphatic.” The medical subject headings (MeSH) used were as follows: indocyanine green, oral cavity neoplasms, and lymph nodes. Duplicate studies were first removed, and the studies were screened systematically based on the title and abstract, followed by a full-text screening. Taking the PubMed database search formula as an example, the search formula is as follows:

((“Oral Cavity Neoplasms”[Mesh]) OR ((((((oral cancer) OR (gingiva cancer)) OR (tongue cancer)) OR (month floor cancer)) OR (lip cancer)) OR (buccal mucosa cancer)) OR (palate cancer))) AND (((“Lymph Nodes”[Mesh]) OR (lymphatic)) OR (LN))) AND ((“Indocyanine Green”[Mesh]) OR (ICG)).

### Inclusion and exclusion criteria

Metastatic LNs were detected via ICG NIR fluorescence in oral cavity cancer in the evaluation index, which contained data regarding true positive (TP), false positive (FP), false negative (FN), and true negative (TN) values. The inclusion and exclusion criteria used in the literature screening were as follows:

1. Inclusion criteria: (i) LN resection for oral cavity cancer and pathological examination as the reference; (ii) ICG-guided SLN mapping; (iii) SLN determined the main research objective; and (iv) the reference standard and diagnostic methods had pathological data, pathologically examined routinely, which is typical of paraffin-embedded sections and immunohistochemical analysis after staining with hematoxylin and eosin (HE).
2. Exclusion criteria: (i) repeated articles, reviews, conference reports, case reports, or letters; (ii) animal studies, ICG photothermal therapy-related studies; (iii) no cervical LN dissection; and (iv) lack of pathological examination or inability to obtain pathological data and the evaluation index (TP, FP, FN, and TN).

### Data extraction

Two investigators independently screened the articles based on the inclusion and exclusion criteria and then assessed the study quality using the Quality Assessment of Diagnostic Accuracy Studies (QUADAS)-2 evaluation criteria. Disagreements between the two investigators were resolved by referring to a third researcher. The investigators extracted the information contained in each study into an Excel spreadsheet, including the first author’s last name, year of publication, study design, country, number of patients, age, type of disease, surgical procedure, total number of LNs collected, total number of fluorescent LNs, positive SLNs, average SLN number obtained in each patient, SLN detection method (ICG, radioisotope, and blue dye), method of pathological detection, commercial name of the ICG, concentration, dose, injection method, injection time, fluorescence imaging and detection time of the technique, follow-up results, and adverse reactions associated with ICG injection.

### Calculation of the review parameters

According to the “PICO” principle, we identified P (population) as a patient with oral cavity cancer (cN_0_), I (intervention) as SLNB guided by ICG near-infrared fluorescence, C (control) as the reference test for diagnosis of postoperative pathological results, and O (outcome) as the diagnosis of metastatic lymph nodes. Therefore, we summarized the detection of SLNB in oral cavity cancer before or during the operation using ICG NIR fluorescence and compared it with the reference test (pathology) to evaluate the accuracy of ICG in detecting metastatic lymph nodes. For each study, TP, TN, PF, and FN rates were estimated accordingly. LNs detected via ICG NIR fluorescence were assessed by calculating the following values:



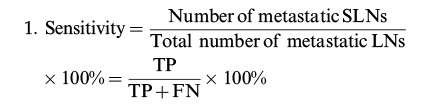





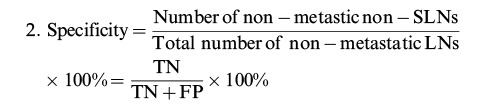





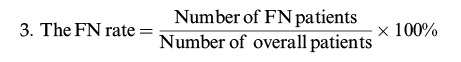





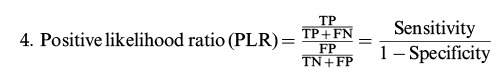





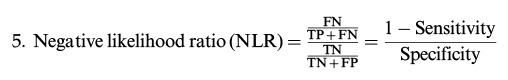





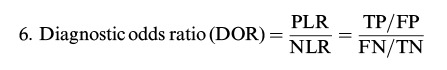



### Statistical analysis

Statistical analyses were performed using Stata version 14.0 (Stata Corp. LLC, College Station, TX, USA) and Meta-Disc version 1.4 (Universitario Ramony Cajal Hospital, Madrid, Spain), which reported the ICG of NIR fluorescence-guided SLNB detection and routine pathological biopsy results of cervical LNs. Study quality was assessed using RevMan software (version 5.3; The Nordic Cochrane Centre, Copenhagen, Denmark). First, Meta-Disc was used to perform Spearman correlation analysis to assess the heterogeneity caused by the threshold effect. A *p*-value>0.05, indicated that there was no heterogeneity caused by the threshold effect while a *p*-value<0.05, indicated the existence of heterogeneity caused by threshold effects. Heterogeneity analysis was performed on sensitivity, specificity, PLR, NLR, and DOR using the Cochran Q test and *I* ^2^ test. A *p*-value<0.05, indicated the existence of significant heterogeneity among the studies, and a random-effects model was used accordingly. A *p*-value>0.05 indicated the absence of significant heterogeneity among the studies, and the fixed-effects model was used for the analysis. A value of *I* ^2^>50% represented high heterogeneity, 50%≥*I^2^*≥25% indicated moderate heterogeneity, and *I* ^2^<25% indicated low heterogeneity. A meta-analysis was performed using a fixed-effects model or random-effects model for heterogeneity, pooling the combined detection rates, summaries of sensitivity, specificity, PLR, NLR, DOR, and summary receiver operating characteristic curve (SROC). Finally, a funnel plot was constructed using Stata to assess publication bias.

## RESULTS

### Characteristics of the studies

**Figure 1:**
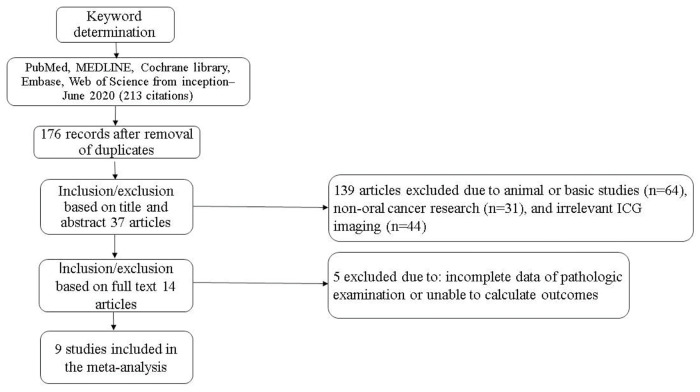
Flowchart of study selection adapted from PRISMA.

**Figure 2:**
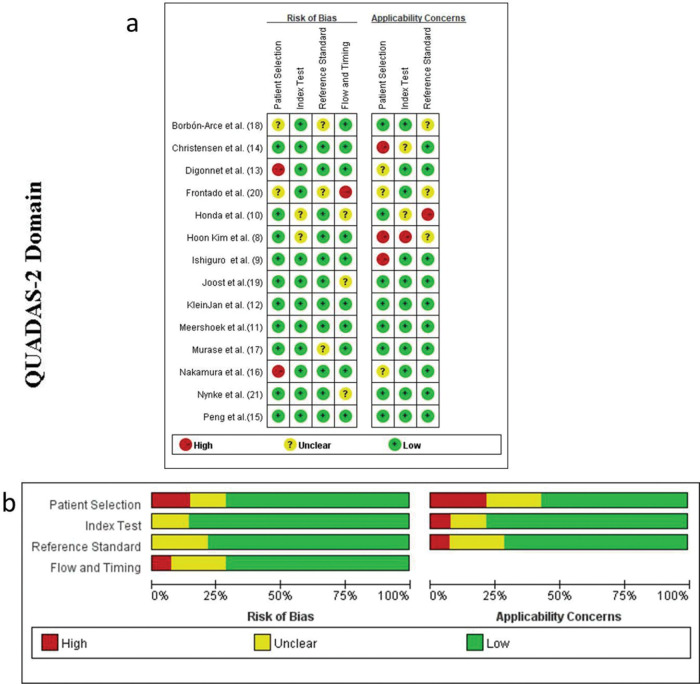
Methodological evaluation of the included studies according to the QUADAS-2 criteria (a) overall and (b) by study QUADAS, Quality Assessment of Diagnostic Accuracy Studies.

A total of 213 records were retrieved based on the search strategy. After removal of duplicates and manual screening for relevant titles and abstracts, the full text of 37 studies was assessed to check for inconsistencies and incomplete data of the research participants. Finally, 14 related studies were included in the systematic review. Of them, nine studies met the relevant criteria and were included in the meta-analysis (8-21) (Figure 1). The included studies subjected to quality evaluation using the QUADAS-2 are shown in Figure 2.

### Patient characteristics and SLNB detection

Fourteen studies involving a total of 226 patients with a mean age of 61.5 (range, 48-65.5) years, were included in this review. The characteristics of these studies are presented in Table 1. ICG-guided fluorescence imaging was used for SLNB detection in each study, and the surgical procedures performed were selective LN dissection in all these studies. The LNs detected were assessed via routine pathological examination as the reference test, typically involving HE staining and immunohistochemistry. A total of 752 SLNs were detected by imaging, with a mean number of 3.33 in each patient, and the combined detection rate was 97%. In the present study, only one patient had lip cancer (13). One case of fluorescent LN was detected by SLNB, and no metastasis was confirmed by pathology.

### Technical aspects of ICG NIR fluorescence

Different commercial labels of ICG and different concentrations (0.25, 1.25 mg/ml, 2.5, and 5 mg/ml) and doses (0.2-2 ml) of the same were used among the included studies. The ICG injection site was peritumoral injection. The time of injection could be preoperative, intraoperative, preoperative, or intraoperative respectively. In addition, other detection methods (^99m^Tc, human serum albumin, and blue dye) were used, with the application of corresponding equipment including the Firefly system, robotic system, computed tomographic lymphography (CTL), photodynamic eye (PDE), fluid-attenuated inversion recovery near-infrared camera (FLAIR), γ-probe, SPECT/CT imaging, a HyperEye Medical System (HEMS) monitor, and a Mini-Fluorescence-Assisted Resection and Exploration imaging system. There were differences in NIR fluorescence detection and imaging time (within 1-2 min, 10 min, 15 min, 20 min, 30 min, and 2 h or more). The specific details are listed in Table 2.

### Fluorescent and pathologic result of LNs

The harvested LNs were examined via routine pathology and the results showed that 55 metastatic fluorescent positive LNs (TP) and 3 fluorescent negative but metastasized LNs (FN) were reported form all the studies. Additionally, 697 cases of nonmetastatic LNs were present in the LNs that showed positive fluorescence (FP).

### Overall sensitivity, specificity, and accuracy of ICG NIR fluorescence

**Figure 3:**
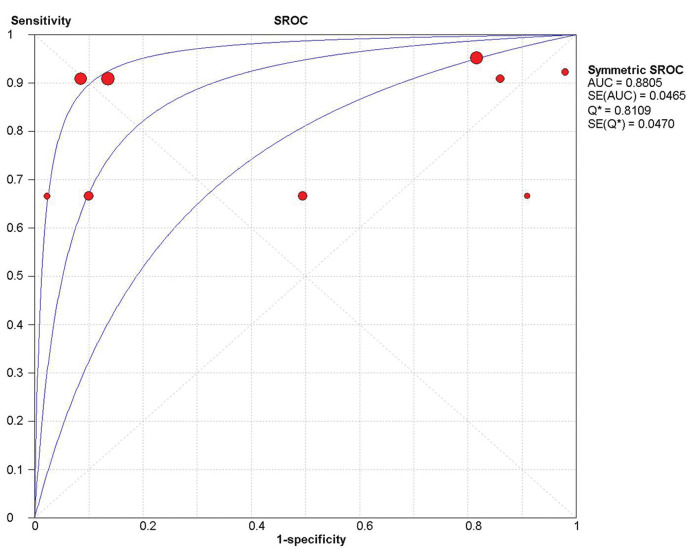
Threshold effect in covariate analysis of sensitivity and specificity of ICG NIR fluorescence imaging among the studies. AUC, area under the curve; ICG, indocyanine green; NIR, near-infrared; SE, standard error; SROC, summary receiver operating characteristic curve.

**Figure 4:**
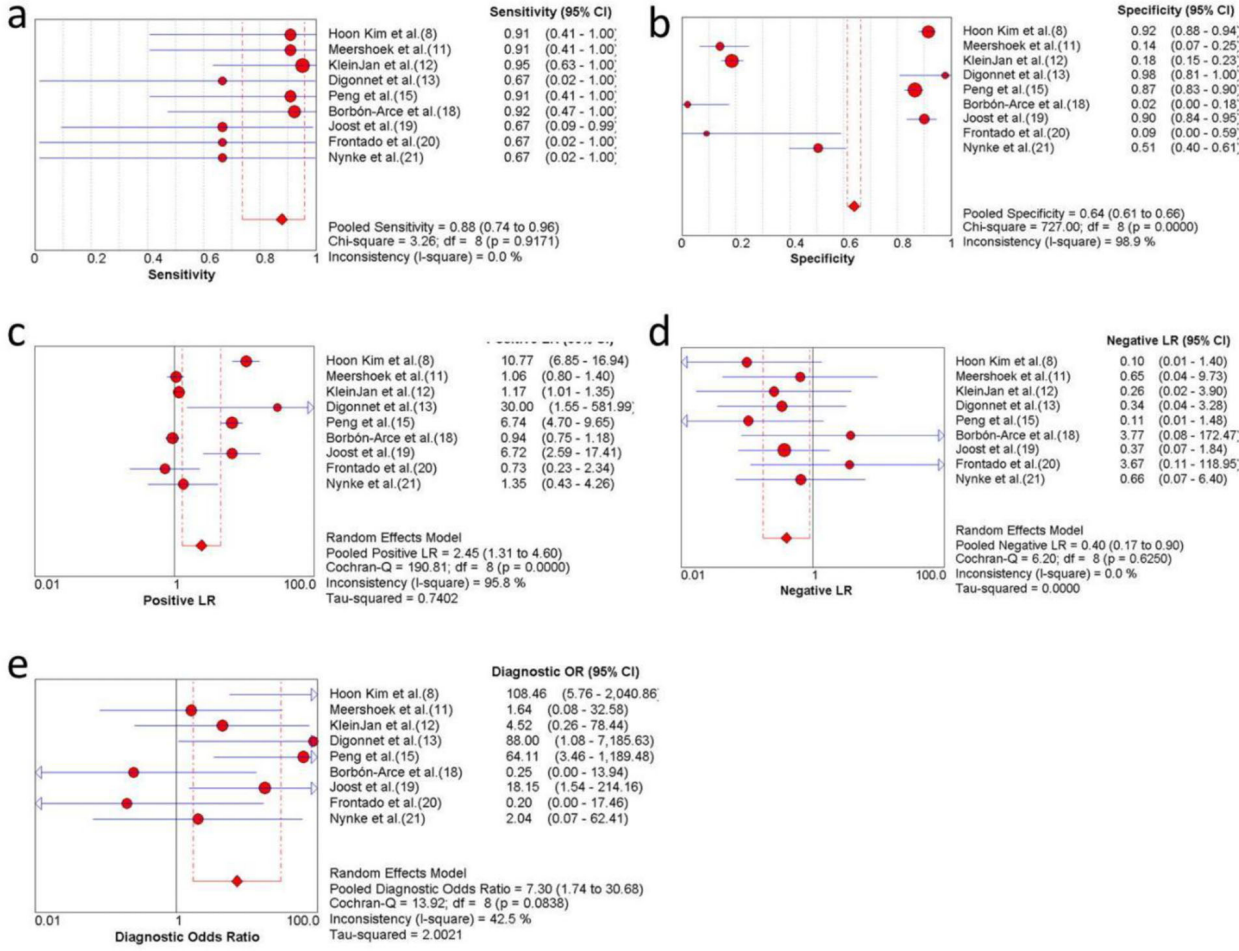
Forest plots of ICG NIR fluorescence imaging in oral cavity cancer (a) sensitivity, (b) specificity, (c) PLR, (d) NLR, and (e) DOR. DOR, Diagnostic odds ratio; ICG, indocyanine green; NIR, near-infrared; NLR, negative likelihood ratio; PLR, Positive likelihood ratio.

**Figure 5:**
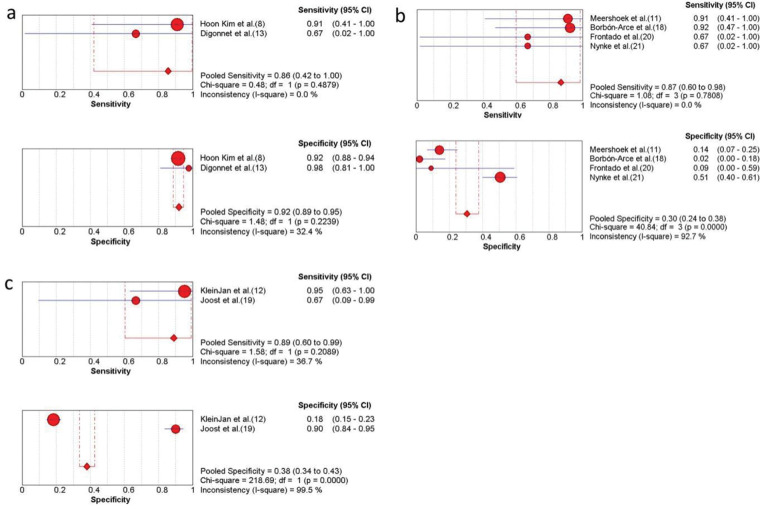
Subgroup analysis of ICG combined with other detection methods. (a) The sensitivity and specificity of only free ICG NIR fluorescence imaging; (b) the sensitivity and specificity of the ICG-^99m^Tc- nanocolloid; (c) the sensitivity and specificity of ICG combined with blue dye and the ICG-^99m^Tc- nanocolloid. CI, confidence interval; ICG, indocyanine green; NIR, near-infrared.

Nine studies were included in the meta-analysis. For the individual and combined incidence rates of FP, FN, TP, and TN, the four grid tables are listed in Table 3. First, the included studies were subjected to threshold effect analysis in Meta-Disc, and the sensitivity logarithm related to the (1-specificity) logarithm of the Spearman coefficient was equal to 0.381 (*p*=0.311, indicating no significance); simultaneously there was no “shoulder arm” distribution in the comprehensive SROC. Both results suggest that there was no threshold effect in this study (Figure 3). All studies were heterogeneously analyzed in Stata 14.0, and the Galbraith plot showed that all points fell within the 95% confidence interval (CI) range. Second, as the analysis in Meta-Disc showed (Figure 5a-e), the median sensitivity for ICG fluorescence in the detection of malignant LNs was 91.0% (95% CI, 67.0-95.0), the median specificity was 51% (95% CI, 2.0-98.0), the median PLR was 1.35 (95% CI, 0.73-30.0), and the median NLR was 0.37 (95% CI, 0.10-3.77). The heterogeneity test for sensitivity as the effect amount yielded *I* ^2^=0.0% (*p*=0.92), indicating little heterogeneity among the studies, and the fixed sensitivity model was set to obtain a pooled sensitivity of 88.0% (95% CI, 74.0-96.0) (Figure 4a). The heterogeneity test for specificity yielded *I* ^2^=98.9% (*p*<0.0001), indicating a large degree of heterogeneity; therefore, a random-effect model analysis was set to obtain a pooled specificity of 64.0% (95% CI, 61.0-66.0) (Figure 4b). Similarly, the pooled PLR and NLR were 2.45 (95% CI, 1.31-4.60) and 0.40 (95% CI, 0.17-0.9), respectively (Figure 4c and d). Regarding the DOR (*I* ^2^=42.5%, *p*=0.084), a fixed-effect model was used to obtain a comprehensive value of 7.30 (95% CI, 1.74-30.68) (Figure 4e). Finally, the area under the combined subject working SROC was 0.88 (*Q* value=0.81), and the standard error was 0.047 (Figure 3).

### Comparison of free ICG and other tracers

As shown in the above results, the pooled sensitivity and specificity of free ICG (n=11) were 86.0% (95% CI, 42.0-100.0) and 92% (95% CI, 89.0-95.0) in three studies (Figure 5a) (8,13). Assessment of ICG-^99m^Tc- nanocolloid (n=45) (11,18,20,21) or the combination of ICG-^99m^Tc- nanocolloid and blue dye (n=59) (12,19) showed that they had a higher sensitivity (Figure 5b, c). It was also concluded from Figure 4 that ICG combined with methylene blue had higher sensitivity (91%) and specificity (87%) (15).

### Assessment of publication bias

A funnel plot constructed in Stata 14.0 to evaluate publication bias yielded a *p*-value of 0.90, indicating no significant publication bias.

### GRADE evidence classification

The GRADE profiler software ranked the evidence quality of the included studies (Table 4). It is thus shown that more than moderate-quality evidence indicates the accuracy of this research.

## DISCUSSION

Occult metastatic LNs are negative on clinical examination but positive when assessed by pathology. Some scholars have assigned them clinically to LN negative (cN_0_) status. A previous study showed that occult LNs were more common in patients with oral cavity cancer and were present in 20%-30% of cases (22). The prognosis of patients with cN_0_ disease with identifiable and dissectible occult metastatic LNs is better than that of patients managed via observation and LN resweeping. However, this approach will also lead to 65%-70% of the cases receiving overtreatment with a wide range of radical neck surgeries in patients with cN_0_ disease (23). Compared with tracers such as radionuclides, ICG is nonradioactive and has a higher biosafety. Additional clinical studies have assessed the application of ICG in SLNB for oral cavity cancer; however, oral cavity cancer has a high rate of LN metastasis and complicated drainage pathways, and the results are not the same according to different reports (24). Therefore, to obtain more objective evidence-based medical support, we conducted a meta-analysis of the diagnostic value of SLNB for oral cavity cancer with ICG as a tracer for metastatic LNs.

During the systematic review, five studies that aimed to detect fluorescent LNs, were evaluated to determine whether neck dissection was performed (9,10,17), and to compare the fluorescence imaging time and fluorescent SLN number according to different SLNB methods (14,16). On account of the lack of comparison with histopathological examination of LNs in neck dissection specimens, these studies were excluded from our final meta-analysis. In this study, for ICG as the main tracer in SLNB, the pooled sensitivity for the detection of metastatic LNs was 88.0% (95% CI, 74.0-96.0), and the median sensitivity was 91.0% (95% CI, 67.0-95.0). The pooled specificity was low at 64.0% (95% CI, 61.0-66.0). Although the highest specificity was 98.0% (95% CI, 81.0-100.0), the median specificity was 51% (95% CI, 2.0-98.0). The reason for the low specificity was that there were six studies with a lower number of fluorescence (-) and metastasis (-) LNs than fluorescent (+) and metastatic (-) SLNs, resulting in a lower TN/TN+FP value. Specifically, the above results may be due to the following reasons: (i) The choice of the surgical procedure, as the selected studies mostly used selective SLN dissection, resulting in postoperative detection of mostly fluorescent (+) SLNs and fewer fluorescence (-) and metastasis (-) LNs (TN). (ii) The choice of clinical cases; the number of patients included in the study was small or the number of LNs with occult metastasis was relatively small, resulting in more fluorescence (+) and metastasis (-) LNs (FP). (iii) The special lymphatic drainage system of the head and neck, making SLNB for oral cavity cancer more complicated than for other cancers (25).

Similar to dyes and radionuclides, ICG injected into the normal tissues surrounding the cancer lesions drains to the LNs along with the lymph. SLNs can then be identified and located via lymphatic scintigraphy. Ramamurthy et al. (26) applied methylene blue dye alone to locate the SLN of oral cancer and reported a recognition rate of 90.6% (n=29/32) and an FN rate of 20%. In this meta-analysis, we included ICG in combination with blue dye or nuclides in the SLNB of oral cavity cancer and performed sensitivity and specificity analyses accordingly. The results showed that the hybrid ICG-^99m^Tc-nanocolloid could increase the sensitivity of metastatic LN detection to 87% (95% CI, 60-96), and there were no FN cases in the limited sample size (11,18,20,21). Similarly, ICG in combination with methylene blue exhibited higher sensitivity than ICG alone (15). However, both specificities were lower, which may be related to the reasons described previously. In addition to the different tracers, different imaging devices also affected the SLN detection results. Taken together, this approach is feasible for SLNB using tracer technology in oral cavity cancer; however, the research evidence needs to be further strengthened.

Other factors may also affect the detection of SLNs in oral cavity cancers. The concentrations of ICG were different (5 mg/ml, 2.5 mg/ml, 1.25 mg/ml, and 0.25 mg/ml), and the detection effect was also different. Using 5 mg/ml ICG (n=22), the sensitivity and specificity of SLN detection were 86% (95% CI, 42-100) and 85% (95% CI, 82-89), respectively; using 2.5 mg/ml ICG (n=40), the sensitivity and specificity were 85% (95% CI, 59-97) and 80% (95% CI, 77-84), respectively; and using 1.25 mg/ml (n=70) yielded a sensitivity and specificity of 94% (95% CI, 70-100) and 18% (95% CI, 14-22), respectively. From the above results, it is seen that the sensitivity of SLN detection also increased with increasing ICG concentration. However, in the study where ICG was applied for the detection of SLNB in gastric cancer, Meifeng et al. (27) found that the sensitivity of the concentration of 5 mg/ml was lower than that of 0.5 or 0.05 mg/ml (0.83 *versus* 0.98), which was related to the study design as mentioned earlier. Therefore, the relationship between ICG concentration and SLN detection sensitivity requires a large sample study to be conducted.

Few studies have examined the postoperative recurrence of SLNB in patients with cancer. Seven studies with existing follow-up records were included in our study, and four of them had no local recurrence or regional LN metastasis occurrence, but one ICG imaging patient died of lung metastasis (17). The remaining three studies reported that at least one patient experienced local tumor recurrence (9,10,12). FN results of SLNB in HNC may miss metastatic LNs, leading to postoperative recurrence. Knackstedt et al. (28) and Ahmed et al. (29) performed ICG-guided SLNB in patients with HNC, with FN rates of 1.64% (1/61) and 20% (4/20), respectively. In this meta-analysis, 226 patients were included, with an FN rate of 0.88% (2/226). The causes of FN were as follows: (i) no micrometastases in SLNs found on pathological examination; (ii) the metastatic tumor cells blocked the draining lymphatic vessels of the primary tumor, causing changes in the drainage pathway and there was a failure to identify true SLNs; and (iii) a metastatic shift in the LNs had occurred (30). Lymphatic drainage is complicated, and SLNs are difficult to locate in the head and neck; therefore, the results regarding the effectiveness of SLNB as applied to oral cavity cancer are inconsistent.

This meta-analysis suggests that ICG-guided SLNB can effectively detect SLN to determine further metastatic LN in patients with oral cavity cancer, especially those with T1 and T2. In the GRADE evidence rating, there is more than moderate-quality evidence that ICG applied to SLNB for the diagnosis of metastatic lymph nodes in oral cavity cancer has high sensitivity; however, the specificity is still greatly affected by the sample size. In the future, it is necessary to expand the sample size and analyze more high-quality studies to evaluate its clinical value.

## CONCLUSIONS

ICG applied to SLNB for detecting regional metastatic LNs was demonstrated to be feasible for oral cavity cancer. Based on the evidence from this study with a limited sample size, ICG combined with dyes or radionuclide-guided SLN mapping increased the sensitivity and specificity and reduced the occurrence of FN results. Only nine studies that were heterogeneous were included in this study. Therefore, further well-designed studies with larger sample sizes are needed to yield more reliable follow-up results.

## AUTHOR CONTRIBUTIONS

Chen Y, Wang Y and Zhang K were responsible for the study design. Xiao Q, Zou W, Xia C and Yin H were responsible for the data acquisition and analysis. Xiao Q, Xia C, Yin H and Pu Y were responsible for the results interpretation, manuscript writing and revision.

## Figures and Tables

**Table 1:** Characteristics of the studies included.

Author	Year	Country	No.	Age (years)	Tumor	Reference test	Procedure	LNs	SLNs	SLN^+^	Mean SLNs	Follow-up
Hoon Kim et al. (8)	2020	South Korea	9	48	OSCC	pathology	RAND	313	31	5	3.4	No recurrence or metastasis were seen
Ishiguro et al. (9)	2020	Japan	27	65	tongue cancer	pathology	SND	ND	41	5	1.5	76 months, 3 of 22 patients without SLN metastasis had occult cervical lymph node metastasis, and 1 SLN positive case recurred
Honda et al. (10)	2019	Japan	16	65.5	tongue cancer	pathology	SND	ND	30	5	1.9	38 months, neck recurrence was found in 2 of the 11 patients who initially showed metastasis-negative SLNs
Meershoek et al. (11)	2019	Netherlands	19	59	tongue cancer	pathology	SND	69	60	5	3.2	-
KleinJan et al. (12)	2018	Netherlands	51	61	OSCC	pathology	SND	357	293	10	5.7	33 months, false negative findings of oral cavity patients occurred in the first 15 cases
Digonnet et al. (13)	2016	Belgium	2	56.5	oral cancer	pathology	SND	23	1	1	-	3-22 months (0 recurrence)
Christensen et al. (14)	2016	Denmark	30	64	OSCC	pathology	SND	ND	94	6	3.1	-
Peng et al. (15)	2015	China	19	60.5	OSCC	pathology	SND (18)RND (1)	420	61	5	3.2	-
Nakamura et al. (16)	2015	Japan	3	-	tongue cancer	pathology	SND	ND	8	1	2.7	No recurrence or metastasis were seen
Murase et al. (17)	2015	Japan	16	65.5	oral cancer	pathology	SND	ND	35	2	2.2	more than 3 years, (1 ICG imaging patient died of lung metastasis; no other signs of recurrence or metastasis were seen)
Borbón-Arce et al. (18)	2014	Spain	9	60	OSCC	pathology	SND	30	30	6	3.3	-
Joost et al. (19)	2013	Germany	8	59.5	oral cancer	pathology	SND	184	15	2	1.9	-
Frontado et al. (20)	2013	Spain	3	52	OSCC	pathology	SND	6	6	1	2	-
Nynke et al. (21)	2012	Netherlands	14	60.5	OSCC	pathology	SND	94	47	1	3.4	-

**Abbreviations:** ND, not detected; OSCC, oral squamous cell cancer; RAND, robotic retroauricular neck dissection; SND, selective neck dissection; SLN, sentinel lymph node SLN^+^, positive SLN.

**Table 2:** Technical aspects of ICG near-infrared fluorescence.

Author	Commercial name of ICG	Concentration	Dose	Detection method	Injection site	Equipment	Injection time	Fluorescent time
Hoon Kim et al. (8)		2.5 mg/ml	2 ml	ICG	peritumoral	Firefly system, robotic system,	preoperative	-
Ishiguro et al. (9)	Daiichi Sankyo Company, Tokyo, Japan	5 mg/ml	2 ml	ICG	peritumoral	CTL, near-infrared camera	intraoperative	-
Honda et al. (10)	Daiichi Pharmaceutical	5 mg/ml	2 ml	CT lymphography, ICG	peritumoral	FLAIR, near-infrared camera, HEMS, PDE	preoperative	1 min/2 min
Meershoek et al. (11)	ICG-Pulsion; Pulsion Medical Systems	1.25 mg/ml	80 MBq	ICG-^99m^Tc-nanocolloid	peritumoral	PDE, near-infrared camera,	preoperative	-
KleinJan et al. (12)	Pulsion Medical Systems, Múnich, Germany	1.25 mg/ml	81 MBq	ICG-^99m^Tc-nanocolloid, blue dye	peritumoral	SPECT/CT, near-infrared camera, γ-probe	preoperative	15 min/2 h
Digonnet et al. (13)	Pulsion Medical System, Belgium	0.25 mg/kg	-	ICG	intravenous	near-infrared camera	intraoperative	2 h
Christensen et al. (14)	ICG-Pulsion,Pulsion Medical, Germany	0.25 mg/ml	0.2 ml	ICG-^99m^Tc -nanocolloid	peritumoral	SPECT/CT, near-infrared camera, γ-probe	preoperative	-
Peng et al. (15)	Dandong, China	5 mg/ml	1 ml	blue dye, ICG	peritumoral	near-infrared camera	preoperative	10 min
Nakamura et al. (16)	Daiichi Sankyo, Tokyo, Japan	2.5 mg/ml	0.5 ml	^99m^Tc, ICG	peritumoral	HEMS monitor,	preoperative	19.8±12.6 min
Murase et al. (17)	Diagnogreen; Daiichi Pharmaceutical, Tokyo, Japan	5 mg/ml	0.4 ml	ICG	peritumoral	γ-probe	intraoperative	2 h
Borbón-Arce et al. (18)	ICG-Pulsion, Pulsion Medical Systems, Múnich, Germany	2.5 mg/ml	85 MBq	ICG-^99m^ Tc-nanocolloid	peritumoral	SPECT/CT, near-infrared camera, γ-probe, PDE	preoperative	15 min/2 h
Joost et al. (19)	Pulsion Medical Systems (Múnich, Germany)	2.5 mg/ml	1.6 mL	blue dye, ICG-^99m^ Tc, HSA	peritumoral	Mini-FLARE imaging system	preoperative	5/10/15/30 min
Frontado et al. (20)	ICG-Pulsion, Pulsion Medical Systems, Múnich, Alemania	5 mg/ml	86.5 MBq	ICG-^99m^ Tc-nanocolloid	peritumoral	SPECT/CT, γ- camera	preoperative	10 min/15 min/2 h
Nynke et al. (21)	Pulsion Medical Systems, Múnich, Germany	2.5 mg/ml	0.4 ml	ICG-^99m^ Tc -nanocolloid	peritumoral	SPECT/CT, near-infrared camera, γ-probe,	preoperative	3-19 h

**Abbreviations:** HEMS, HyperEye Medical System; CT, computed tomography; CTL, computed tomographic lymphography; FLAIR, fluid-attenuated inversion recovery; ICG, indocyanine green; PDE, Photo Dynamic Eye; SPECT, single-photon emission computed tomography.

**Table 3:** The TP, FP, FN, and TN results of the included articles.

Author	TP	FP	FN	TN
Hoon Kim et al. (8)	5	26	0	282
Meershoek et al. (11)	5	55	0	9
KleinJan et al. (12)	10	283	0	64
Digonnet et al. (13)	1	0	0	22
Peng et al. (15)	5	56	0	359
Borbón-Arce et al. (18)	6	24	0	0
Joost et al. (19)	2	13	1	118
Frontado et al. (20)	1	5	0	0
Nynke et al. (21)	1	46	0	47
Total	36	508	1	901

**Abbreviations:** FN, false positive, FN, false negative; TP, true positive, TN, true negative.

**Table 4:** Sensitivity and specificity GRADE classification results of SLNB in HNC using indocyanine green.

				Factors that may decrease certainty of evidence					
Study	Outcome	No. of patients	Study design	Risk of bias	Indirectness	Inconsistency	Imprecision	publication bias	Test accuracy CoE
Hoon Kim et al. (8)	sensitivity	9	cross-sectional	not serious	not serious	not serious	not serious	not serious	⊕⊕⊕○/B
	specificity		(-1)^a^	not serious	not serious	not serious	not serious	not serious	⊕⊕⊕○/B
Meershoek et al. (11)	sensitivity	19	cross-sectional	not serious	not serious	not serious	not serious	not serious	⊕⊕⊕○/B
	specificity		(-1)^a^	not serious	not serious	(-1)^b^	not serious	not serious	⊕⊕○○/C
KleinJan et al. (12)	sensitivity	51	cross-sectional	not serious	not serious	(+1)	not serious	not serious	⊕⊕⊕⊕/A
	specificity		(-1)^a^	not serious	not serious	not serious	not serious	not serious	⊕⊕⊕○/B
Digonnet et al. (13)	Sensitivity	2	cross-sectional	not serious	not serious	not serious	(-1)^c^	not serious	⊕⊕○○/C
	specificity		(-1)^a^	not serious	not serious	not serious	(-1)^c^	not serious	⊕⊕○○/C
Peng et al. (15)	Sensitivity	19	cross-sectional	not serious	not serious	not serious	not serious	not serious	⊕⊕⊕○/B
	specificity		(-1)^a^	not serious	not serious	not serious	not serious	not serious	⊕⊕⊕○/B
Borbón-Arce et al. (18)	Sensitivity	9	cross-sectional	not serious	not serious	not serious	not serious	not serious	⊕⊕⊕○/B
	specificity		(-1)^a^	not serious	not serious	(-1)^b^	not serious	not serious	⊕⊕○○/C
Joost et al. (19)	Sensitivity	8	cross-sectional	not serious	not serious	not serious	not serious	not serious	⊕⊕⊕○/B
	specificity		(-1)^a^	not serious	not serious	not serious	not serious	not serious	⊕⊕⊕○/B
Frontado et al. (20)	Sensitivity	3	cross-sectional	not serious	not serious	not serious	(-1)^c^	not serious	⊕⊕○○/C
	specificity		(-1)^a^	not serious	not serious	not serious	(-1)^c^	not serious	⊕⊕○○/C
Nynke et al. (21)	Sensitivity	14	cross-sectional	not serious	not serious	not serious	not serious	not serious	⊕⊕⊕○/B
	specificity		(-1)^a^	not serious	not serious	not serious	not serious	not serious	⊕⊕⊕○/B

**Abbreviations:** HNC, head and neck cancer; SLNB, sentinel lymph node biopsy. **a** cross-sectional study is not a randomized controlled study, and the level of evidence needs to be downgraded; **b.** the sample has low homogeneity, considered to degrade; **c)** the size of homogeneous sample affects the reliability of the results.
